# Impact of combined ^18^F-FDG PET/CT in head and neck tumours

**DOI:** 10.1038/sj.bjc.6602464

**Published:** 2005-03-15

**Authors:** R Syed, J B Bomanji, N Nagabhushan, S Hughes, I Kayani, A Groves, S Gacinovic, N Hydes, D Visvikis, C Copland, P J Ell

**Affiliations:** 1 Institute of Nuclear Medicine, Middlesex Hospital, Mortimer Street, London W1N 3AA, UK; 2 Department of Radiology, St Georges Hospital, London, UK; 3 Belfast & Department of Maxillofacial Surgery, St Georges Hospital, London, UK

**Keywords:** ^18^F-FDG PET/CT, imaging, head and neck cancers, squamous cell carcinoma

## Abstract

To compare the interobserver agreement and degree of confidence in anatomical localisation of lesions using 2-[fluorine-18]fluoro-2-deoxy-D-glucose (^18^F-FDG) positron emission tomography (PET)/computed tomography (CT) and ^18^F-FDG PET alone in patients with head and neck tumours. A prospective study of 24 patients (16 male, eight female, median age 59 years) with head and neck tumours was undertaken. ^18^F-FDG PET/CT was performed for staging purposes. 2D images were acquired over the head and neck area using a GE Discovery LS™ PET/CT scanner. ^18^F-FDG PET images were interpreted by three independent observers. The observers were asked to localise abnormal ^18^F-FDG activity to an anatomical territory and score the degree of confidence in localisation on a scale from 1 to 3 (1=exact region unknown; 2=probable; 3=definite). For all ^18^F-FDG-avid lesions, standardised uptake values (SUVs) were also calculated. After 3 weeks, the same exercise was carried out using ^18^F-FDG PET/CT images, where CT and fused volume data were made available to observers. The degree of interobserver agreement was measured in both instances. A total of six primary lesions with abnormal ^18^F-FDG uptake (SUV range 7.2–22) were identified on ^18^F-FDG PET alone and on ^18^F-FDG PET/CT. In all, 15 nonprimary tumour sites were identified with ^18^F-FDG PET only (SUV range 4.5–11.7), while 17 were identified on ^18^F-FDG PET/CT. Using ^18^F-FDG PET only, correct localisation was documented in three of six primary lesions, while ^18^F-FDG PET/CT correctly identified all primary sites. In nonprimary tumour sites, ^18^F-FDG PET/CT improved the degree of confidence in anatomical localisation by 51%. Interobserver agreement in assigning primary and nonprimary lesions to anatomical territories was moderate using ^18^F-FDG PET alone (kappa coefficients of 0.45 and 0.54, respectively), but almost perfect with ^18^F-FDG PET/CT (kappa coefficients of 0.90 and 0.93, respectively). We conclude that ^18^F-FDG PET/CT significantly increases interobserver agreement and confidence in disease localisation of ^18^F-FDG-avid lesions in patients with head and neck cancers.

Squamous cell carcinoma is the most common tumour type in head and neck cancers (Ak *et al*, 2000). At presentation, 40% of patients have localised disease, while 60% have advanced malignancies (Ak *et al*, 2000). Cervical lymph nodes are the most common site for tumour spread (Mendenhall *et al*, 1998; Ak *et al*, 2000), and this is a relatively common clinical presentation (Mendenhall *et al*, 1998; Ak *et al*, 2000). The presence or absence of cervical lymphadenopathy is of prognostic significance (Myers *et al*, 1998).

The role of 2-[fluorine-18]fluoro-2-deoxy-D-glucose (^18^F-FDG) positron emission tomography (PET) imaging has previously been evaluated in head and neck tumours, the reported sensitivity and specificity varying between 90 and 96% in primary and treated disease (Myers *et al*, 1998). Determining the precise location of ^18^F-FDG-avid lesions by PET alone can be challenging in these patients, as the test suffers from poor anatomical localisation that might compromise sensitivity (Ak *et al*, 2000). The recent introduction of combined ^18^F-FDG PET and computed tomography (CT) imaging has revolutionised imaging by allowing accurate anatomical localisation of functional abnormalities. However, no large trial has as yet confirmed this advantage in head and neck tumours.

The aim of this pilot study was to assess the degree of interobserver agreement and confidence in anatomical localisation of primary head and neck tumours and their metastases with ^18^F-FDG PET alone and with ^18^F-FDG PET/CT.

## MATERIALS AND METHODS

### Patient group

Between June 2000 and August 2003, 24 patients with histologically proven head and neck tumours who underwent whole-body ^18^F-FDG PET/CT for staging were included in a prospective study. This study, examining the imaging strength of ^18^F-FDG PET/CT, is part of an ongoing prospective trial looking at the role of ^18^F-FDG PET scintigraphy in head and neck cancer. The study was approved by the local ethics committee.

### Inclusion criteria

The inclusion criteria were: histologically confirmed diagnosis of head and neck cancer and absence of prior chemotherapy, radiotherapy or surgery to the head and neck region.

### Patient preparation

All patients went through a standard protocol with a minimum of 6 h fasting prior to the study in order to minimise glucose utilisation by normal tissue. Patients were given a muscle relaxant just before the injection (5 mg diazepam orally) to reduce muscle uptake. Blood glucose level was checked prior to ^18^F-FDG injection, and if it was less than 10 mmol l^−1^, the patient was intravenously injected with ^18^F-FDG (mean injected activity=380 MBq, range 370–400 MBq) calibrated just before the injection. Patients rested for 45 min before imaging and were advised to remain silent to reduce skeletal muscle uptake.

### DATA acquisition and reconstruction

PET/CT was performed using the GE Advance PET scanner and the GE Light-speed multislice spiral CT. The Light-speed CT acquires four 5-mm slices at 140 kV with 80 mA and a large pitch of 6 (30 mm of table travel per gantry rotation).

### CT protocol

Four-detector multislice CT images were acquired using speed of rotation and couch movement of 0.8 s and 30 mm per rotation, respectively. The images were reconstructed in 4.25 mm slice width during normal respiration. CT images were rebinned from a 512 × 512 matrix to a 128 × 128 matrix and matched to the pixel size of the PET data in order to match the in-slice resolution of the PET emission images. The CT images were subsequently converted to maps of PET attenuation coefficients using a bilinear transformation based on the use of different scaling factors for materials with Hounsfield units (HU) ⩽0 and >0.

### PET protocol

Without changing the patient position, a whole-body PET emission scan was performed over the same area as was covered by CT (five to six bed positions). All acquisitions were carried out in 2D mode, the protocol comprising an emission scan with 5 min per bed position. PET images were reconstructed using CT attenuation maps. Transaxial emission images of 4.3 × 4.3 × 4.25 mm^3^ (in plane matrix size 128 × 128, 35 slices per bed position) were reconstructed using ordered subsets expectation maximisation (OSEM) with two iterations and 28 subsets. The axial field of view was 148.75 mm, with acquisition of 35 slices per bed position.

### Analysis

Two nuclear medicine physicians and one radiologist (blinded to the results of clinical and other radiological findings) were informed that they were evaluating studies in patients with head and neck cancers. Each reporter interpreted the studies independently. The ^18^F-FDG PET scans were reviewed first and the ^18^F-FDG PET/CT images were interpreted 3 weeks later.

The readers were asked to identify abnormal sites of increased uptake and assign a score as follows:

- Activity at or above brain cerebral cortex: score of 3.
- Activity between that of brain and liver: score of 2.
- Activity any less than b: score of 1.

Lesions were considered positive if they had an ^18^F-FDG uptake score of at least 2. These areas were also quantitatively assessed with measurements (standard uptake values (SUVs)) using region of interest (ROI) analysis. Irregular ROIs were drawn over the most avid part of the identified lesion, the margin being identified visually on a grey scale of 0–20 000 Bq ml^−1^. Mean and maximal values of the ^18^F-FDG uptake were determined.

Inter-reader variability for all of the reporting tasks outlined was assessed using kappa statistics: kappa values of >0.8, 0.61–0.8 and 0.41–0.6 represented perfect, substantial and moderate agreement, respectively (Cohen, 1960; Landis and Koch, 1977; Fleiss, 1981). The confidence in anatomically localising the primary lesion was also assessed. Areas of abnormal increase in ^18^F-FDG activity were assigned to an anatomical territory, and the degree of confidence in anatomical localisation was scored on a scale of 1–3 as follows:

- Exact anatomical region doubtful: score of 1.
- Probable anatomical localisation: score of 2.
- Definite anatomical localisation: score of 3.

## RESULTS

The study group included 16 males and eight females with a median age of 59 years (range 36–86 years). Of the 24 patients, 12 had squamous cell carcinoma of the aerodigestive tract (located in the hypopharynx in eight cases and in the oropharynx in four), as confirmed by the postoperative surgical specimen. In the remaining patients, no primary lesion was identified but nodal disease was confirmed histologically (as metastatic squamous cell carcinoma deposits).

### Abnormal activity on PET

Of the 24 patients, 18 had positive and six negative ^18^F-FDG PET scans. In the aforementioned 18 patients, 21 ^18^F-FDG-avid lesions were identified, of which six were primary tumours (SUV range 7.2–22) and 15 were nonprimary lesions (SUV range 4.5–11.7).

### Anatomical localisation: PET only

^18^F-FDG PET identified the anatomical site correctly in only 12 of the 21 ^18^F-FDG-avid lesions (57%): three (25%) were primary tumour sites and nine (75%) were sites of nonprimary lesions. No specific anatomical sites could be identified in nine of the 21 lesions (43%), comprising three primary and six nonprimary lesions.

### Anatomical localisation: PET/CT

^18^F-FDG PET/CT images identified 21 ^18^F-FDG-avid lesions in 18 patients. Of these 21 lesions, six were primary lesions and 15 were nonprimary lesions. Of the 15 nonprimary lesions, two were located in the neck muscles, two in fat planes (a normal variant), nine at nodal sites, one in lung and one in a rib (Table 1). Enhanced CT identified two additional lesions, which showed no ^18^F-FDG uptake and which were subsequently confirmed to be benign enlarged cervical nodes on histology.

### Lesion-based analysis

The total number of lesions identified by each observer using ^18^F-FDG PET alone and ^18^F-FDG PET/CT was similar. The interobserver variability in assigning FDG-avid lesions to an anatomical territory when using ^18^F-FDG PET alone and ^18^F-FDG PET/CT is shown in Table 2. Using ^18^F-FDG PET/CT, the confidence in anatomical localisation improved by 57% for observer 1, 43% for observer 2 and 52% for observer 3 (Table 3).

Using ^18^F-FDG PET alone, correct anatomical localisation was documented in 50% of primary lesions and 60% of nonprimary lesions. Using ^18^F-FDG PET/CT, confidence in assigning lesions to an anatomical territory improved by 50% in primary sites and 51% in nonprimary sites.

### Interobserver agreement

The calculated interobserver agreement for the three observers in identifying ^18^F-FDG-avid primary and nonprimary sites was almost perfect (100%) with both ^18^F-FDG PET alone and ^18^F-FDG PET/CT. However, when using ^18^F-FDG PET alone, there was only moderate agreement between the three observers in assigning primary and nonprimary lesions to anatomical territories, with kappa coefficients of 0.45 and 0.54, respectively (Table 4). On the other hand, there was strong agreement between all three observers in assigning both primary and nonprimary lesions to anatomical territories when using ^18^F-FDG PET/CT, with kappa coefficients of 0.90 and 0.93, respectively (Table 4).

## DISCUSSION

**Figure 1:**
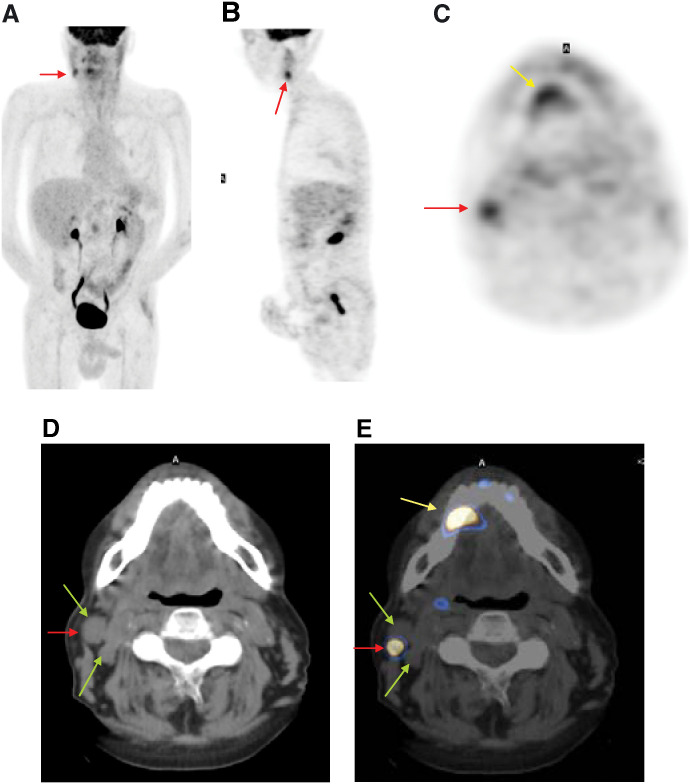
A 50-year-old male with squamous cell carcinoma of the tongue. (**A**) Multiple intensity projection ^18^F-FDG PET image. (**B**) Sagittal and (**C**) transaxial images show abnormal uptake in the right cervical region and right premolar area; dental pathology is present (yellow arrow). (**D**) CT transaxial section reveals level II enlarged lymph nodes. (**E**) Fused ^18^F-FDG PET/CT transaxial section at the same level reveals that one lymph node is FDG positive (red arrow), while the other nodes shows no avidity for FDG (green arrows). The fused images clearly localised the exact site of involvement.

This study shows the advantage of ^18^F-FDG PET/CT over ^18^F-FDG PET alone in disease localisation in patients with head and neck cancers. Our data demonstrate strong interobserver agreement in lesion localisation between the three readers on ^18^F-FDG PET/CT but not on ^18^F-FDG PET alone. The study also shows that ^18^F-FDG PET/CT improves the confidence in assigning lesions to an anatomical territory by 50% in primary and 51% in nonprimary sites, compared with ^18^F-FDG PET alone (Figure 1).

**Figure 2:**
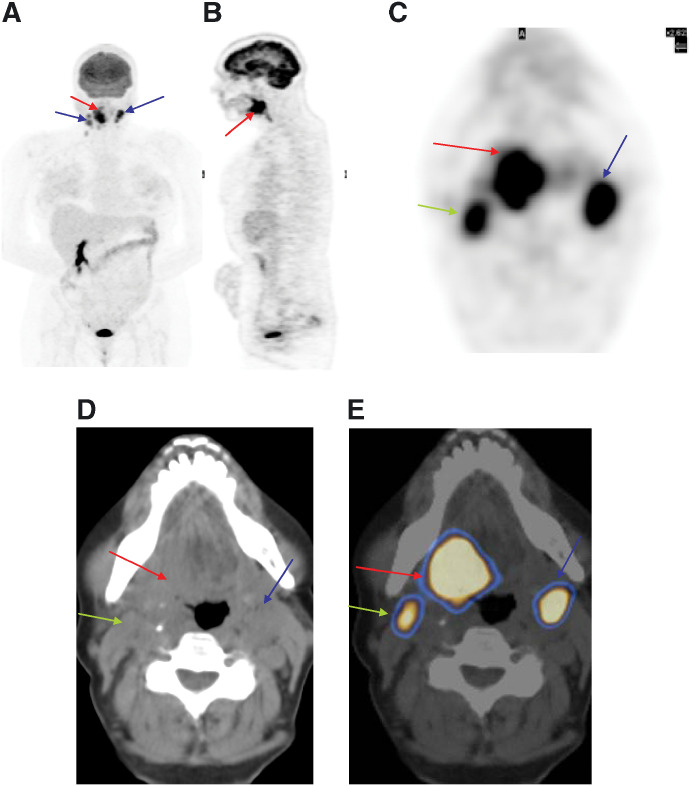
A 42-year-old male with squamous cell carcinoma of tongue. (**A**) ^18^F-FDG PET/CT multiple intensity projection image shows the primary site (red arrow) with bilateral cervical nodes (blue arrows). (**B**) Sagittal and (**C**) transaxial images show abnormal uptake in the known primary (posterior part of the tongue) and both cervical regions. (**D**) CT transaxial section reveals a lesion in the base of the tongue along with left cervical node enlargement. (**E**) Fused ^18^F-FDG PET/CT transaxial section at the same level reveals the exact anatomical site of ^18^F-FDG uptake in the right side of the tongue base extending across the midline and level II left cervical lymph node. The ^18^F-FDG activity in the right cervical region correlates to the right sternocleidomastoid muscle (green arrow) (a normal variant).

These results are of clinical significance in the head and neck territory, where the anatomy is complex. Use of ^18^F-FDG PET alone suffers from lack of anatomical outline identification, which makes precise localisation difficult. In turn, this renders image analysis difficult, and may give rise to false-positive findings. We observed four false-positive sites in our study as follows: in four patients, false-positive lesions were identified in the neck on ^18^F-FDG PET alone. These were asymmetrical in distribution and were considered positive nodal metastases; however, ^18^F-FDG PET/CT clearly localised these to brown fat planes and muscle uptake (Table 1). It is well known that nonspecific uptake in brown fat planes and muscle attachment sites (Figure 2E) is a common cause of false-positive results (Engel *et al*, 1996; Kostakoglu *et al*, 1996; McGuirt *et al*, 1998). This false-positive feature has a critical impact on nodal staging of head and neck tumours (Helmberger *et al*, 1996; Myers *et al*, 1998; Hannah *et al*, 2002). The possibility of false-positive sites on ^18^F-FDG PET alone also holds true in the postsurgical and postradiotherapy neck, where distortion of normal anatomy can further add to the complexity. In this context, the ability to fuse ^18^F-FDG uptake to an anatomical structure defined on CT carries a significant advantage in reducing false-positive results. In our study, ^18^F-FDG PET/CT downstaged the disease in the aforementioned cases, and the management was changed from wide neck dissection to local radiotherapy and local surgery.

In our hands, ^18^F-FDG PET/CT demonstrated a sensitivity of 91% and a specificity of 93% in identifying disease. Similar results for PET/CT have been reported previously (Meltzer *et al*, 2001). For ^18^F-FDG PET, Laubenbacher *et al* (1995) reported a sensitivity of 90% and a specificity of 96%, Bailet *et al* (1992) a sensitivity of 71% and a specificity of 98% and Braams *et al* (1995) a sensitivity of 91% and a specificity of 88%.

In this study, we correctly identified nine ^18^F-FDG-avid metastatic cervical lymph nodes (seven (78%) ipsilateral and two (22%) contralateral). In addition, two non-nodal metastases were identified (one in lung and one in rib). These latter two lesions were reported with both ^18^F-FDG PET and ^18^F-FDG PET/CT, but accurate anatomical localisation was only possible with ^18^F-FDG PET/CT (Table 1). Subsequently, both patients received palliative treatment for symptom relief. This highlights the advantage of whole-body ^18^F-FDG PET in identifying distant metastases (M staging). The ability to detect unexpected contralateral neck disease, second primary tumours and distant metastases by whole-body ^18^F-FDG PET/CT imaging has clear implications and may dramatically alter treatment planning, with the emphasis shifting to a less aggressive approach. Thus, it is important that whole-body ^18^F-FDG PET/CT scanning is performed in these patients to detect distant metastases, and that imaging should not be restricted to the head and neck area because of time constraints.

^18^F-FDG PET/CT also identified the primary lesion in two patients who presented with metastatic neck disease and in whom the primary could not be identified despite conventional investigations. The ability to detect the primary with ^18^F-FDG PET/CT has significant management implications (Kluetz *et al*, 2000). In such cases, definitive treatment can be instituted at the primary site, rather than irradiating potential sites empirically or, as is the practice at some centres, instituting an expectant policy and managing the primary site if and when it becomes clinically evident. Furthermore, by pinpointing the areas of involvement, the surgeon can better define margins and spare structures that are not affected by malignancy.

Finally, although not part of this particular study, another aspect of patient management where combined PET/CT can be beneficial is in the investigation of recurrent disease and evaluation of response to therapy. This can be exceptionally difficult with conventional imaging alone, and is currently managed by repeated EUA/endoscopy and ‘best guess biopsy’. Such an approach is often distressing and morbid for the patient and has significant resource implications.

Our data further highlight the advantage of ^18^F-FDG PET/CT and show identical interobserver agreement in lesion detection and localisation between the three readers on ^18^F-FDG PET/CT but not on ^18^F-FDG PET alone. This was despite the variation in speciality (nuclear medicine and radiology) of the readers. It might be argued that this improvement was subjective and was biased by the observer's knowledge of the type of scan used. However, the significant improvement in interobserver agreement between the three observers refutes the above line of reasoning and serves to validate the argument that the improved confidence among the three observers was a reflection of improved anatomical localisation by ^18^F-FDG PET/CT in real terms.

Some limitations of this study need to be mentioned. The small size of the patient cohort limited accurate assessment of sensitivity and specificity of ^18^F-FDG PET/CT. Furthermore, the cohort under study were treatment naive; after surgery and radiotherapy, different rates of detection of residual or recurrent disease would be expected with ^18^F-FDG PET/CT and ^18^F-FDG PET alone.

## CONCLUSION

- ^18^F-FDG PET alone suffers from poor anatomical localisation of head and neck cancers and their metastases.
- ^18^F-FDG uptake at sites of brown fat/muscle attachment can mimic cervical nodal metastases on ^18^F-FDG PET alone but not on ^18^F-FDG PET/CT.
- ^18^F-FDG PET/CT demonstrates strong interobserver agreement and improves confidence in anatomical localisation of ^18^F-FDG-avid disease by approximately 50%.

## Figures and Tables

**Table 1:** Comparison between ^18^F-FDG PET alone and ^18^F-FDG PET/CT in identifying ^18^F-FDG-avid primary and nonprimary lesions

	**Primary lesions**	**Nonprimary lesions**				
		**Lymph nodes^a^**	**Fat uptake**	**Muscle**	**Lung**	**Rib**
^18^F-FDG PET	6	13	Hot spot reported (unable to define anatomical territory)	Hot spot reported (unable to define anatomical territory)	1 Hot spot reported (unable to define anatomical territory)	1 Hot spot reported (unable to define anatomical territory)
						
^18^F-FDG PET/CT	6	9	2	2	1 Confirmed on high-resolution CT	1 Confirmed on bone scan

^18^F-FDG PET=2-[fluorine-18]fluoro-2-deoxy-D-glucose; CT=computed tomography.

a Includes four lesion identified as activity in the lymph nodes on ^18^F-FDG PET and were subsequently confirmed to be fat and muscle uptake on ^18^F-FDG PET/CT.

**Table 2:** Interobserver variability in assigning ^18^F-FDG-avid lesions to an anatomical territory using ^18^F-FDG PET alone and ^18^F-FDG PET/CT

	**Confidence score for anatomical localisation of lesions**	**No. of FDG-avid lesions with each score/total no. of FDG-avid lesions**	
		**^18^F-FDG PET alone**	**^18^F-FDG PET/CT**
Observer 1	1	6/21	2/21
	2	5/21	1/21
	3	10/21	18/21
			
Observer 2	1	2/21	0/21
	2	4/21	4/21
	3	15/21	17/21
			
Observer 3	1	1/21	0/21
	2	3/21	2/21
	3	17/21	19/21

^18^F-FDG PET=2-[fluorine-18]fluoro-2-deoxy-D-glucose; CT=computed tomography.

**Table 3:** Improvement in confidence of each observer in assigning ^18^F-FDG-avid lesions to an anatomical territory when using ^18^F-FDG PET/CT, as compared with ^18^F-FDG PET alone

	**^18^F-FDG PET alone**		**^18^F-FDG PET/CT**		
	**Lesion detectability**	**Anatomical localisation**	**Lesion detectability^a^**	**Anatomical localisation^a^**	**(%) Improve- ment**
Observer 1	21	12	23	23	57
Observer 2	21	9	23	23	43
Observer 3	21	11	23	23	52
		51%		100%	51

^18^F-FDG PET=2-[fluorine-18]fluoro-2-deoxy-D-glucose; CT=computed tomography.

a Includes two lesions identified on enhanced CT, which did not show ^18^F-FDG uptake and were subsequently confirmed to be benign enlarged cervical nodes on histology.

**Table 4:** Kappa coefficient with 95% confidence intervals between three observers using ^18^F-FDG PET alone and ^18^F-FDG PET/CT

	**^18^F-FDG PET alone**		**^18^F-FDG PET/CT**	
	**Kappa coefficient**	**95% Confidence interval**	**Kappa coefficient**	**95% Confidence interval**
Site of primary	1	0.88–1.12	1	0.88–1.11
Localisation of tumour	0.45	0.27–0.62	0.90	0.82–1.02
No. of nodes	1	0.9–1.09	1	0.9–1.09
Localisation of nodes	0.54	0.31–0.72	0.93	0.89–1.06

^18^F-FDG PET=2-[fluorine-18]fluoro-2-deoxy-D-glucose; CT=computed tomography.

## References

[bib1] Ak I, Stokkel MP, Pauwels EK (2000) Positron emission tomography with 2-(^18^F) fluoro-2-deoxy-D-glucose in oncology. Part II. The clinical value in detecting and staging primary tumours. J Cancer Res Clin Oncol 126: 560–574 11043393 10.1007/PL00008466 PMC12165135

[bib2] Bailet JW, Abeymayor E, Jabour BA, Hawkins RA, Ho C, Ward PH (1992) Positron emission tomography. A new, precise imaging modality for detection of primary head and neck tumours and assessment of cervical adenopathy. Laryngoscope 102: 281–288 1545657 10.1288/00005537-199203000-00010

[bib3] Braams JW, Pruim J, Freling NJ, Nikkels PG, Roodenburg JL, Boering G, Vaalburg W, Vermey A (1995) Detection of lymph node metastases of squamous-cell cancer of the head and neck with FDG-PET and MRI. J Nucl Med 36: 211–216 7830116

[bib4] Cohen J (1960) A coefficient of agreement for nominal scales. Educ Psychol Meas 20: 37–46

[bib5] Engel H, Steinert H, Buck A, Berthold T, Huch Boni RA, Von Sculthess GK (1996) Whole-body PET: physiological and artifactual fluorodeoxyglucose accumulation. J Nucl Med 37(3): 441–446 8772641

[bib6] Fleiss JL (1981) The Measurement of Interrater Agreement. Statistical Methods for Rates and Proportions, 2nd edn pp 212–304. New York: John Wiley & Sons Inc.

[bib7] Hannah A, Scott AM, Tochon-Danguy H, Chan JG, Akhurst T, Berlangieri S, Price D, Smith GJ, Schelleman T, McKay WJ, Sizeland A (2002) Evaluation of ^18^F-fluorodeoxyglucose positron emission tomography and computed tomography with histopathologic correlation in the initial staging of head and neck cancer. Ann Surg 236(2): 208–217 12170026 10.1097/00000658-200208000-00009 PMC1422567

[bib8] Helmberger R, Jager L, Grevers G, Reiser M (1996) Computerized tomography of malignancies of the oral cavity, the oropharynx and hypopharynx and invasiveness. Radiologe 36: 193–198 8693081 10.1007/s001170050060

[bib9] Kluetz PG, Meltzer CC, Villemagne VL, Kinahan PE, Chander S, Martinelli MA, Townsend DW (2000) Combined PET/CT imaging in oncology. Impact on patient management. Clin Positron Imaging 3(6): 223–230 11378434 10.1016/s1095-0397(01)00055-3

[bib10] Kostakoglu L, Wong JC, Barrington SF, Cronin BF, Dynes AM, Maisey MN (1996) Speech-related visualization of laryngeal muscles with fluorine-18-FDG. J Nucl Med 37(11): 1771–1773 8917172

[bib11] Landis JR, Koch GG (1977) The management of observer agreement for categorical data. Biometrics 33: 159–174 843571

[bib12] Laubenbacher C, Saumweber D, Wagner-Manslau C, Kau RJ, Herz M, Avril N, Ziegler S, Kruschke C, Arnold W, Schwaiger M (1995) Comparison of fluorine-18-fluorodeoxyglucose PET, MRI, endoscopy for staging head and neck squamous-cell carcinomas. J Nucl Med 36: 1747–1757 7562038

[bib13] McGuirt WF, Greven K, Williams III D, Keyes Jr JW, Watson N, Cappellari JO, Geisinger KR (1998) PET scanning in head and neck oncology: a review. Head Neck 20: 208–215 9570626 10.1002/(sici)1097-0347(199805)20:3<208::aid-hed5>3.0.co;2-4

[bib14] Meltzer CC, Snyderman CH, Fukui MB, Bascom DA, Chander S, Johnson JT, Myer EN, Martinelli MA, Kinahan PE, Townsend DW (2001) Combined PET/CT imaging in head and neck cancer: impact on patient management. J Nucl Med 42: 36 (abstr)

[bib15] Mendenhall WM, Mancuso AA, Parsons JT, Stringer SP, Cassisi NJ (1998) Diagnostic evaluation of squamous cell carcinoma metastatic to cervical lymph nodes from unknown head and neck primary site. Head Neck 20: 739–744 9790297 10.1002/(sici)1097-0347(199812)20:8<739::aid-hed13>3.0.co;2-0

[bib16] Myers LL, Wax WK, Nabi H, Simpson GT, Lamonica D (1998) Positron emission tomography in the evaluation of the N0 neck. Laryngoscope 108: 232–236 9473074 10.1097/00005537-199802000-00014

